# Effects of drivers and their variations on the number of stems and aboveground carbon removals in miombo woodlands of mainland Tanzania

**DOI:** 10.1186/s13021-021-00180-9

**Published:** 2021-05-19

**Authors:** Bernardol John Manyanda, Emmanuel F. Nzunda, Wilson Ancelm Mugasha, Rogers Ernest Malimbwi

**Affiliations:** Department of Forest Resources Assessment and Management, College of Forestry, Wildlife and Tourism, Sokoine University of Agriculture, Chuo Kikuu, P.O. Box 3013, Morogoro, Tanzania

**Keywords:** Drivers, Aboveground carbon, Emissions, miombo, Removals

## Abstract

**Background:**

Removals caused by both natural and anthropogenic drivers such as logging and fire in miombo woodlands causes substantial carbon emissions. Here we present drivers and their effects on the variations on the number of stems and aboveground carbon (AGC) removals based on an analysis of Tanzania’s national forest inventory (NFI) data extracted from the National Forest Resources Assessment and Monitoring (NAFORMA) database using allometric models that utilize stump diameter as the sole predictor.

**Results:**

Drivers of AGC removals in miombo woodlands of mainland Tanzania in order of importance were timber, fire, shifting cultivation, charcoal, natural death, firewood collection, poles, grazing by wildlife animals, carvings, grazing by domestic animals, and mining. The average number of stems and AGC removals by driver ranged from 0.006 to 16.587 stems ha^−1^ year^−1^ and 0.0–1.273 tCha^−1^ year^−1^ respectively. Furthermore, charcoal, shifting cultivation and fuelwood caused higher tree removals as opposed to timber, natural death and fire that accounted for higher AGC removals.

**Conclusions:**

Drivers caused substantial effects on the number of stems and carbon removals. Increased mitigation efforts in addressing removals by timber, fires, shifting cultivation, charcoal and natural death would be effective in mitigating degradation in miombo woodlands of Tanzania. Additionally, site-specific studies need to be conducted to bring information that would be used for managing woodlands at local levels. This kind of study need to be conducted in other vegetation types like montane and Mangrove forest at national scale in Tanzania.

## Background

Managing the carbon stocks of the land use sector is currently a key focus for climate change mitigation in developing countries [1–3]. In terrestrial ecosystems, forests and woodlands play a major role for the mitigation and adaptation to climate change via carbon storage [2, 4]. After oceans, forests and woodlands are the world’s largest storehouses of carbon and they provide ecosystem services that are important to human wellbeing [5]. Tropical forests alone store a quarter of a trillion tons of carbon in above and below ground biomass [6]. Notwithstanding their contribution to the climate change mitigation, Tanzania’s forests face enormous challenges including deforestation and forest degradation [7].

Deforestation and forest degradation are amongst the major anthropogenic sources of greenhouse gas emissions (GHG), contributing about 17 per cent globally [8]. Of the total emissions, degradation is responsible for at least one-fifth in the Brazilian Amazon [9], two-thirds in Indonesian forests [10], and almost half in African tropical forests [11]. Forest degradation also leads to forest fragmentation and can contribute to deforestation [12]. While deforestation refers to a permanent or long-term conversion of forest to non-forest land [13, 14], forest degradation is the changes within the forest that negatively affect the structure or function of the stand and/or site, and thereby lower the capacity to supply products and/or services [15, 16].

The changes within the forests involves removals of trees and hence contributing to carbon emissions. The drivers of carbon removals are multifaceted and cannot be reduced to a few variables; rather they operate at different levels and scales in the human–environment linkage [17]. These drivers are divided into two broad categories: proximate and underlying causes. Proximate causes are typically human activities operating at the local level. They include shifting cultivation and cattle ranching, wood extraction through logging or charcoal production, and infrastructural development such as transportation, markets and settlements. On the other hand, underlying causes do not directly cause removals but influence the proximate causes. This category includes a complexity of economic issues, policies and institutions, technological factors, socio-cultural, and demographic factors [11, 17, 18].

Aboveground biomass (AGB) is not static, but rather spatially and temporally highly variable, particularly in the tropics with the same factor likely having different results [19–21]. This makes its quantification challenging. Carbon stocks are widely estimated from forest biomass estimates [22]. Many authors assume the carbon concentration of tree in different vegetation types including miombo woodlands to be between 45 and 50% of the dry biomass [22–25]. Miombo woodlands are the largest vegetation types in Tanzania covering about 93% of the forest area of 48.1million ha [26]. As in other tropical forest landscape, complex matrices of low to high AGC removal densities can be expected in entire miombo woodlands in Tanzania and its management categories due to varying drivers. Additionally, which drivers contribute more to the variations of AGC removals in the entire miombo woodlands and its management categories is largely unknown. This has been due to lack of appropriate assessment mechanism. Nevertheless, the NFI data source which is commonly referred as NAFORMA, have recently become available based on country Reduced emission from deforestation and forest degradation plus the roles of conservation, sustainable management of forests and enhanced carbon stocks (REDD+) readiness activities that allow assessment of drivers of AGC removals and their amount of AGC emissions in miombo woodlands [3]. The objective of the present study was to identify the drivers of AGC removals and assess which of the identified drivers contribute more to the variation of AGC in miombo woodlands of Tanzania mainland. Specifically the study sought to: (1) identify drivers of AGC removals (2) quantify the amount of AGC removals by each driver and, (3) Ranking the identified drivers in order of their contribution to the variations of AGC.

Understanding the drivers of AGC removals and their amount of AGC removed is fundamental for better design of REDD+ strategy. In some cases, REDD+ incentives would be channeled directly to affect drivers. Moreover, a better understanding of drivers of AGC removals are required as part of developing mitigation interventions at sub-national levels to ensure improved land-use change. This kind of understanding is also crucial for subsequent development of management plans in order to tackle each driver in response to the amount of AGC emissions caused.

## Results

We identified eleven drivers for tree cutting i.e. forest fires, firewood collection, grazing by both wildlife, domesticated animals, carving, poles, shifting cultivation, timber, and mining activities (Table 1). Furthermore, the contribution of the drivers in terms of the number of stems and AGC removals per hectare per year for miombo woodlands in Tanzania is also shown in Table 1. Higher number of stems/ha/year were removed by shifting cultivation, followed by charcoal, natural death, firewood collection and poles. In terms of carbon however, we observed higher AGC removals by timber followed by fire, shifting cultivation, charcoal and natural death (Table 1).

**Table 1:** Drivers and their corresponding number of stems and AGC removals in mainland Tanzania

Drivers	Stems ha^−1^ yr^−1^	Stems % ha^−1^ yr^−1^	AGB tha^−1^ yr^−1^	AGC tCha^−1^ yr^−1^	AGC % tCha^−1^ yr^−1^
Timber	0.780	7.000	0.244	0.119	20.173
Fire	0.845	7.581	0.196	0.096	16.235
Shifting cultivation	2.741	24.595	0.191	0.093	15.788
Charcoal	1.747	15.672	0.182	0.089	15.085
Natural death	1.233	11.061	0.160	0.079	13.268
Firewood collection	1.376	12.343	0.089	0.043	7.331
Poles	1.588	14.250	0.078	0.038	6.494
Unknown	0.472	4.233	0.047	0.023	3.871
Grazing wild	0.256	2.294	0.014	0.007	1.192
Carvings	0.050	0.450	0.004	0.002	0.350
Grazing domestic	0.053	0.471	0.002	0.001	0.203
Mining	0.006	0.051	0.000	0.000	0.010

The contribution of the drivers concerning number of stems and carbon removals were further expressed based on different management categories and subcategories of miombo woodlands. Considering Tanzania Forest Services Agency (TFS) administrative zones, large number of stems were removed by charcoal followed by firewood collection and shifting cultivation whereas grazing was the least in the central zones (Table 2). On the other hand, charcoal removed more AGC followed by firewood collection, natural death and shifting cultivation while grazing had the least removals. Regarding eastern zone, charcoal took higher number of stems per hectare per year followed by firewood, natural death, timber, shifting cultivation and poles while the least was grazing domestic. In contrast, timber accounted for higher carbon removals followed by charcoal, natural death, shifting cultivation and firewood collection in the eastern zone. The effects of the drivers on number of stems and carbon removals in other zones seem to change leading positions between charcoal production, timber, natural death, shifting cultivation and fire (Table 2).

**Table 2:** Drivers and their corresponding number of stems and AGC removals in zones of mainland Tanzania

Zone names	Drivers	Stems ha^−1^ yr^−1^	Stems % ha^−1^ yr^−1^	AGB tha^−1^ yr^−1^	AGC tCha^−1^ yr^−1^	AGC % tCha^−1^ yr^−1^
Central	Charcoal	3.687	28.472	0.187	0.092	49.202
	Firewood collection	3.653	28.213	0.055	0.027	14.429
	Timber	1.334	10.303	0.046	0.023	12.215
	Shifting cultivation	1.808	13.961	0.042	0.020	10.969
	Natural death	0.693	5.351	0.027	0.013	7.131
	Fire	0.870	6.722	0.018	0.009	4.662
	Poles	0.820	6.332	0.003	0.001	0.787
	Grazing domestic	0.019	0.148	0.001	0.001	0.380
	Grazing wild	0.065	0.498	0.001	0.000	0.225
	Total	12.949	100	0.380	0.186	100
Eastern	Timber	5.812	12.466	0.746	0.366	30.732
	Charcoal	11.014	23.625	0.655	0.321	26.979
	Natural death	7.904	16.954	0.405	0.198	16.670
	Shifting cultivation	5.151	11.050	0.189	0.092	7.772
	Firewood collection	8.157	17.497	0.178	0.087	7.337
	Poles	5.137	11.019	0.097	0.048	4.004
	Fire	1.022	2.193	0.079	0.039	3.258
	Grazing wild	2.201	4.722	0.069	0.034	2.844
	Carvings	0.199	0.426	0.010	0.005	0.396
	Grazing domestic	0.023	0.048	0.000	0.000	0.008
	Total	46.62	100	2.428	1.19	100
Lake	Fire	0.515	6.604	0.089	0.044	33.115
	Poles	2.303	29.520	0.049	0.024	18.096
	Firewood collection	2.555	32.755	0.042	0.021	15.674
	Timber	0.379	4.862	0.035	0.017	13.031
	Charcoal	0.663	8.495	0.023	0.011	8.555
	Natural death	0.782	10.023	0.023	0.011	8.720
	Shifting cultivation	0.417	5.347	0.005	0.003	1.901
	Grazing wild	0.187	2.394	0.002	0.001	0.909
	Total	7.801	100	0.268	0.132	100
Northern	Charcoal	8.040	21.454	0.331	0.162	22.579
	Shifting cultivation	13.459	35.915	0.324	0.159	22.145
	Timber	1.257	3.355	0.301	0.147	20.525
	Poles	6.117	16.325	0.209	0.102	14.248
	Natural death	4.459	11.898	0.166	0.081	11.301
	Firewood collection	2.881	7.688	0.103	0.05	7.006
	Fire	1.092	2.915	0.030	0.015	2.072
	Grazing wild	0.168	0.449	0.002	0.001	0.124
	Total	37.473	100	1.466	0.717	100
Southern highlands	Natural death	2.397	25.501	0.332	0.163	59.949
	Poles	3.026	32.2	0.08	0.039	14.403
	Timber	0.649	6.908	0.075	0.037	13.576
	Firewood collection	2.294	24.407	0.052	0.026	9.44
	Shifting cultivation	0.621	6.61	0.008	0.004	1.401
	Grazing domestic	0.091	0.964	0.004	0.002	0.731
	Charcoal	0.258	2.741	0.002	0.001	0.421
	Grazing wild	0.063	0.67	0	0	0.079
	Total	9.399	100	0.553	0.272	100
Southern	Fire	4.143	14.368	0.586	0.287	36.115
	Timber	2.149	7.455	0.304	0.149	18.729
	Shifting cultivation	7.009	24.309	0.293	0.144	18.049
	Natural death	7.122	24.7	0.183	0.09	11.257
	Poles	4.477	15.528	0.112	0.055	6.923
	Charcoal	0.885	3.07	0.064	0.031	3.916
	Firewood collection	1.54	5.342	0.048	0.024	2.961
	Grazing wild	0.884	3.067	0.019	0.01	1.199
	Carvings	0.311	1.078	0.011	0.006	0.702
	Grazing domestic	0.233	0.809	0.002	0.001	0.121
	Mining	0.079	0.273	0	0	0.028
	Total	28.832	100	1.622	0.797	100
Western	Timber	1.782	11.582	0.163	0.08	27.946
	Shifting cultivation	5.317	34.564	0.127	0.062	21.766
	Natural death	1.592	10.348	0.073	0.036	12.584
	Firewood collection	1.866	12.128	0.065	0.032	11.195
	Charcoal	1.364	8.866	0.059	0.029	10.053
	Fire	0.922	5.993	0.059	0.029	10.169
	Poles	2.419	15.726	0.031	0.015	5.366
	Grazing domestic	0.025	0.163	0.005	0.002	0.794
	Grazing wild	0.097	0.632	0.001	0	0.128
	Total	15.384	100	0.583	0.285	100

Considering vegetation types, natural death, timber production and shifting cultivation appear to be leading causes of removals interchangeably in the closed woodlands, open woodlands and woodlands with scattered cropland for both number of stems and AGB respectively (Table 3). Grazing, mining and carvings are among the least contributors to removals in the three vegetation types. Regarding ownership types, higher number of stem removals were observed due to natural death followed by fire, grazing wild, poles and timber in the central government land (Table 4). While the least number of stems removals per hectare per year was observed due to carving followed by grazing domestic and charcoal. The highest AGC was removed as timber followed by natural death, fire and charcoal. Carvings, grazing domestic and shifting cultivation accounted for the least AGC removals in this ownership types (Table 4). In the local government owned woodlands; firewood collection took higher number of stems per hectare per year followed by charcoal, natural death and timber while grazing wild was the least. However, the leading position changed for carbon removal in which Natural death accounted for higher removals followed by Charcoal, timber, and Firewood collection while the least was the same driver (Table 4).

**Table 3:** Drivers and their corresponding number of stems and AGC removals in miombo woodlands vegetation subtypes of mainland Tanzania

Vegetation types	Drivers	Stems ha^−1^ yr^−^	Stems % ha^−1^ yr^−1^	AGB tha^−1^ yr^−1^	AGC tC ha^−1^ yr^−1^	AGC % tC ha^−1^ yr^−1^
Closed woodlands (crown cover > 40%)	Natural death	2.070	28.528	0.251	0.123	26.309
	Timber	0.847	11.680	0.250	0.122	26.236
	Shifting cultivation	0.782	10.782	0.183	0.090	19.174
	Unknown	0.384	5.297	0.069	0.034	7.243
	Fire	0.642	8.843	0.063	0.031	6.657
	Poles	1.041	14.350	0.044	0.022	4.662
	Firewood collection	0.675	9.297	0.040	0.020	4.219
	Charcoal	0.414	5.707	0.040	0.020	4.192
	Grazing wild	0.338	4.658	0.010	0.005	1.091
	Carvings	0.027	0.366	0.001	0.001	0.122
	Grazing domestic	0.036	0.492	0.001	0.000	0.094
	Total	7.255	100	0.953	0.467	100
Open woodlands (Crown cover between 10–40%)	Timber	11.005	51.300	0.262	0.128	20.443
	Fire	0.903	4.210	0.246	0.121	19.225
	Charcoal	1.454	6.779	0.193	0.095	15.070
	Natural death	1.686	7.860	0.167	0.082	13.061
	Shifting cultivation	2.448	11.409	0.158	0.078	12.360
	Firewood collection	1.550	7.226	0.101	0.049	7.882
	Poles	1.538	7.168	0.086	0.042	6.702
	Unknown	0.499	2.328	0.042	0.021	3.297
	Grazing wild	0.245	1.142	0.017	0.008	1.288
	Carvings	0.055	0.258	0.005	0.003	0.419
	Grazing domestic	0.061	0.284	0.003	0.002	0.241
	Mining	0.008	0.037	0.000	0.000	0.013
	Total	21.453	100	1.282	0.628	100
Woodlands with scattered cropland	Shifting cultivation	16.587	58.998	0.735	0.360	47.938
	Charcoal	1.751	6.228	0.241	0.118	15.713
	Timber	0.382	1.360	0.180	0.088	11.715
	Firewood collection	2.032	7.228	0.127	0.062	8.259
	Poles	4.994	17.764	0.121	0.059	7.914
	Natural death	0.858	3.051	0.073	0.036	4.759
	Fire	0.912	3.244	0.039	0.019	2.541
	Unknown	0.452	1.606	0.011	0.005	0.700
	Pole	0.063	0.225	0.006	0.003	0.383
	Carvings	0.083	0.297	0.001	0.001	0.080
	Total	28.114	100	1.533	0.751	100

**Table 4:** Drivers and their corresponding number of stems and AGC removals in ownership types of miombo woodlands in Mainland Tanzania

Ownership types	Drivers	Stems ha^−1^ yr^−^	Stems % ha^−1^ yr^−1^	AGB tha^−1^ yr^−1^	AGC tCha^−1^ yr^−1^	AGC % tCha^−1^ yr^−1^
Central Government	Timber	0.507	10.511	0.114	0.056	26.306
	Natural death	1.503	31.143	0.103	0.050	23.651
	Fire	0.695	14.409	0.079	0.039	18.124
	Charcoal	0.284	5.888	0.037	0.018	8.604
	Grazing wild	0.561	11.617	0.037	0.018	8.467
	Firewood collection	0.331	6.851	0.026	0.013	6.040
	Poles	0.551	11.406	0.020	0.010	4.652
	Shifting cultivation	0.372	7.704	0.016	0.008	3.792
	Carvings	0.011	0.218	0.001	0.000	0.232
	Grazing domestic	0.012	0.254	0.001	0.000	0.132
	Total	4.827	100	0.434	0.213	100
Local Government	Natural death	1.684	23.388	0.310	0.152	33.885
	Charcoal	1.738	24.134	0.217	0.106	23.743
	Timber	0.988	13.719	0.210	0.103	23.032
	Firewood collection	2.315	32.141	0.140	0.068	15.288
	Fire	0.225	3.128	0.031	0.015	3.446
	Shifting cultivation	0.118	1.634	0.004	0.002	0.471
	Poles	0.106	1.476	0.001	0.000	0.080
	Grazing domestic	0.019	0.269	0.000	0.000	0.054
	Grazing wild	0.008	0.111	0.000	0.000	0.002
	Total	7.201	100	0.914	0.448	100
Village land	Fire	1.000	7.911	0.315	0.154	22.305
	Timber	0.841	6.654	0.278	0.136	19.694
	Natural death	2.000	15.815	0.227	0.111	16.082
	Charcoal	1.363	10.779	0.177	0.087	12.550
	Shifting cultivation	3.038	24.024	0.160	0.079	11.366
	Poles	2.337	18.479	0.122	0.060	8.678
	Firewood collection	1.755	13.882	0.115	0.056	8.156
	Grazing wild	0.170	1.347	0.008	0.004	0.564
	Carvings	0.066	0.522	0.004	0.002	0.300
	Grazing domestic	0.063	0.499	0.004	0.002	0.288
	Mining	0.011	0.087	0.000	0.000	0.017
	Total	12.645	100	1.411	0.691	100
Private Land	Shifting cultivation	11.128	59.113	1.102	0.540	60.139
	Charcoal	2.466	13.101	0.355	0.174	19.388
	Timber	0.625	3.320	0.135	0.066	7.344
	Firewood collection	1.913	10.160	0.116	0.057	6.348
	Poles	1.991	10.577	0.059	0.029	3.216
	Natural death	0.374	1.987	0.046	0.023	2.517
	Fire	0.278	1.478	0.016	0.008	0.872
	Pole	0.033	0.175	0.003	0.001	0.167
	Grazing wild	0.016	0.088	0.000	0.000	0.009
	Total	18.825	100	1.832	0.898	100
General land	Timber	1.577	13.068	0.807	0.395	48.146
	Natural death	2.719	22.537	0.247	0.121	14.754
	Charcoal	2.163	17.925	0.226	0.111	13.470
	Fire	1.911	15.838	0.155	0.076	9.268
	Shifting cultivation	1.308	10.839	0.086	0.042	5.127
	Poles	0.854	7.080	0.085	0.042	5.059
	Carvings	0.256	2.124	0.034	0.017	2.056
	Firewood collection	0.721	5.976	0.027	0.013	1.586
	Grazing wild	0.260	2.153	0.006	0.003	0.344
	Grazing domestic	0.297	2.461	0.003	0.002	0.191
	Total	12.065	100	1.675	0.821	100
Unknown	Firewood collection	1.051	68.394	0.010	0.005	69.823
	Natural death	0.486	31.606	0.004	0.002	30.177
	Total	1.536	100	0.014	0.007	100

On the other hand, shifting cultivation accounted for the highest number of stems per hectare per year followed by poles, natural death and firewood collection, charcoal and fire in the village owned woodlands. However, the leading position on the contribution to carbon removals changed in which fire led the position followed by timber, natural death, charcoal, shifting cultivation, poles, firewood collection (Table 4). Considering private land, shifting cultivation removed more stems per hectare per year followed by charcoal, poles and firewood. Interestingly shifting cultivation followed by charcoal removed more carbon as well (Table 4). Furthermore, natural death, took higher number of stems per hectare per year followed by charcoal, fire, timber and shifting cultivation. However, the leading position changed in terms of carbon removal whereby timber led the position followed by natural death, charcoal, fire and shifting (Table 4).

Table 5 indicate drivers and the corresponding number of stem and carbon removals in the different land use types. Regarding protection forestland, the highest number of stems removed were due to natural death followed by fire, grazing wild, timber, firewood collection and poles (Table 5). In terms of AGC, the highest AGC were removed as natural death followed by timber, fire and grazing wild. Grazing by domestic animals, carvings and grazing by wild animals accounted for the least AGC removals in protection forest. Regarding wildlife reserve, natural death led the contribution to both number of stems and carbon removals per hectare per year. While fire and grazing by wild animals was the next leading position from natural death for stems removals, timber and fire was the leading position from natural death for carbon removals (Table 5). In other land use types such as production forest, grazing land, shifting cultivation and water bodies/swamps, drivers of number of stems and carbon removals appear to be changing leading positions (Table 5).

**Table 5:** Drivers and their corresponding number of stems and biomass removals in land use types of miombo woodlands in mainland Tanzania

Ownership types	Drivers	Stems ha^−1^ yr^−^	Stems % ha^−1^ yr^−1^	AGB tha^−1^ yr^−1^	AGC tC ha^−1^ yr^−1^	AGC % tC ha^−1^ yr^−1^
Production forest	Fire	1.144	10.354	0.390	0.191	26.726
	Timber	1.086	9.831	0.380	0.186	26.061
	Charcoal	1.426	12.903	0.203	0.099	13.925
	Natural death	2.294	20.764	0.167	0.082	11.473
	Poles	1.968	17.815	0.108	0.053	7.385
	Firewood collection	1.582	14.318	0.106	0.052	7.284
	Shifting cultivation	0.668	6.046	0.048	0.024	3.313
	Unknown	0.450	4.077	0.031	0.015	2.104
	Grazing wild	0.230	2.082	0.010	0.005	0.717
	Carvings	0.097	0.874	0.010	0.005	0.653
	Grazing domestic	0.089	0.810	0.005	0.002	0.339
	Mining	0.014	0.127	0.000	0.000	0.020
	Total	11.049	100	1.458	0.714	100
Protection forest	Natural death	3.749	40.101	0.176	0.086	44.792
	Timber	0.936	10.012	0.090	0.044	22.917
	Fire	1.754	18.761	0.069	0.029	15.104
	Grazing wild	1.567	16.761	0.045	0.022	11.458
	Poles	0.457	4.888	0.010	0.005	2.604
	Firewood collection	0.671	7.177	0.008	0.004	2.083
	Charcoal	0.083	0.888	0.004	0.002	1.042
	Unknown	0.052	0.556	0.002	0.000	0.000
	Grazing domestic	0.061	0.652	0.001	0.000	0.000
	Shifting cultivation	0.019	0.203	0.000	0.000	0.000
	Total	9.349	100	0.405	0.192	100
Wildlife reserve	Natural death	1.985	38.544	0.213	0.104	40.532
	Timber	0.364	7.079	0.121	0.059	23.125
	Fire	0.903	17.534	0.079	0.039	15.010
	Grazing wild	0.932	18.091	0.060	0.029	11.398
	Unknown	0.282	5.473	0.021	0.010	4.080
	Poles	0.184	3.572	0.014	0.007	2.605
	Firewood collection	0.367	7.131	0.010	0.005	1.992
	Charcoal	0.069	1.343	0.005	0.003	0.994
	Grazing domestic	0.052	1.005	0.001	0.001	0.235
	Shifting cultivation	0.012	0.229	0.000	0.001	0.030
	Total	5.149	100	0.525	0.257	100
Shifting cultivation	Shifting cultivation	21.885	61.492	1.664	0.815	61.559
	Timber	0.985	2.768	0.310	0.152	11.486
	Charcoal	2.368	6.652	0.187	0.092	6.930
	Firewood collection	2.463	6.920	0.150	0.073	5.543
	Poles	4.520	12.700	0.134	0.066	4.973
	Unknown	1.471	4.133	0.118	0.058	4.382
	Natural death	0.952	2.675	0.094	0.046	3.462
	Fire	0.870	2.445	0.044	0.022	1.630
	Carvings	0.077	0.216	0.001	0.000	0.035
	Total	35.591	100	2.703	1.324	100
Agriculture	Shifting cultivation	12.914	49.747	0.884	0.433	46.721
	Firewood collection	4.776	18.399	0.411	0.201	21.685
	Charcoal	2.107	8.116	0.217	0.107	11.484
	Unknown	1.173	4.519	0.105	0.051	5.547
	Fire	0.807	3.107	0.093	0.046	4.932
	Timber	0.405	1.559	0.072	0.035	3.817
	Poles	2.669	10.282	0.065	0.032	3.447
	Natural death	0.962	3.706	0.036	0.018	1.906
	Pole	0.068	0.261	0.006	0.003	0.332
	Grazing domestic	0.034	0.130	0.002	0.001	0.101
	Grazing wild	0.045	0.174	0.001	0.001	0.027
	Total	25.959	100	1.893	0.928	100
Grazing land	Grazing domestic	1.830	17.790	0.606	0.297	43.763
	Charcoal	2.339	22.742	0.410	0.201	29.570
	Shifting cultivation	2.754	26.783	0.101	0.049	7.280
	Poles	1.405	13.657	0.080	0.039	5.767
	Firewood collection	1.281	12.454	0.078	0.038	5.652
	Fire	0.297	2.888	0.055	0.027	3.944
	Timber	0.153	1.492	0.037	0.018	2.669
	Unknown	0.171	1.667	0.014	0.007	0.985
	Carvings	0.017	0.162	0.003	0.001	0.201
	Natural death	0.022	0.216	0.002	0.001	0.121
	Grazing wild	0.015	0.149	0.001	0.001	0.049
	Total	10.284	100	1.385	0.679	100
Built up area	Firewood collection	7.798	100	0.319	0.156	100
	Total	7.798	100	0.319	0.156	100
Water body/swamp	Timber	12.758	66.667	2.599	1.273	95.994
	Poles	6.379	33.333	0.108	0.053	4.006
	Total	19.137	100	2.707	1.327	100
Other land	Fire	1.371	27.861	0.085	0.042	35.878
	Poles	0.415	8.435	0.045	0.022	19.128
	Firewood collection	0.605	12.289	0.041	0.020	17.176
	Natural death	1.102	22.398	0.033	0.016	13.793
	Charcoal	1.105	22.464	0.031	0.015	13.159
	Grazing wild	0.138	2.808	0.001	0.001	0.558
	Shifting cultivation	0.184	3.744	0.001	0.001	0.309
	Total	4.920	100	0.238	0.116	100

## Discussion

The overall objective of this paper was to identify the drivers of AGC removals and to quantify the contributions of each driver to the variation of AGC removals and hence carbon emissions in miombo woodlands in Tanzania using NAFORMA data set. In this study, effects of drivers on AGC and number of stems removals have been reported. The carbon stored in the AGB pool is typically the largest among the Intergovernmental Panel for Climate Change (IPCC) carbon pools for REDD+ reporting purposes. It is understood that while removals by shifting cultivation fire, firewood collection and charcoal, results immediately into carbon emissions, it is not the case with removals for timber, carvings and poles which may end up in construction and furniture whose emissions may be delayed. Nonetheless, timber in the form of furniture, carvings or construction is more in the process of contributing to emissions although delayed. Due to the uncertainty of time taken for timber to act as stored carbon all removals are assumed to eventually to contribute to emissions.

Eleven drivers contributed number of stems and AGC removals in mainland Tanzania. These drivers included charcoal, wildfire, firewood collection, grazing by both wildlife and domesticated animals, carving, poles, shifting cultivation, timber, and mining activities. Since drivers of AGC removals are similar to drivers for forest degradation in the woodlands meant for both protective and production purposes, comparison across studies were based on studies conducted to determine forest degradation drivers. The result found in the present study is comparable to results found in miombo woodland in Masito forest in western Tanzania and Liwale district southern Tanzania [27–29]. These studies documented only six drivers responsible for forest degradation. Sites specific and the methodologies applied on these studies explains fewer documentation of drivers. On the other hand, Carandang et al. [30] documented ten drivers for forest degradation in Philippines that agrees with results from the present study. The methodology employed, particularly on the sampling procedures could explain the similarity.

In terms of the contribution of drivers on the number of stems and AGC removals nationally, removals by shifting cultivation, natural death, poles and charcoal production account for the highest number of stem removals. The reason could be attributed by high demand of charcoal in the country for cooking energy in which small diameter trees are involved. Tanzania’s annual consumption of charcoal is 1,658,000 tons [31]. About 85% of the total urban population depends on charcoal for household cooking and energy for small and medium enterprises [32]. Additionally, more than 40% of the tree removals can be attributed to charcoal use alone in Tanzania [33]. Higher removals by shifting cultivation is probably due to intensification of shifting cultivation in Tanzania. Shifting cultivation in Tanzania occupies 7.6% of the total country land area and 33% of area classified as woodlands in Tanzania [26]. Other scholars [34, 35] asserted that shifting cultivation contribute more to forest degradation due to rising demand for agricultural products, dietary changes, agricultural trade and adjustment. Firewood collection and poles on the other hand, rank third and fourth in taking large amount of stems in the woodlands. This is probably because; firewood is the main source of energy rural areas [36]. The same author noted that, lack of alternative and affordable sources of energy dependence of communities on forests. Construction purposes both in the rural and urban areas probably account for higher removals of trees as poles. Furthermore, climate change impacts like diseases eruptions and severe drought naturally kill trees. These effects have recently increased tremendously. Mining and grazing by domesticated animals appeared as the least drivers responsible for stem removals. This is because of the smallest area subjected into mining and carvings activities.

In terms of AGC removals, timber and fire accounts for the highest AGC removals. This may be explained by the large tree removals that comprises of the largest biomass. According to [37], large trees tend to account for a large proportion of the AGB in mature forests; often between 30 and 40% of the AGB can be found in trees with diameters greater than 70 cm. Elsewhere in miombo, Dewees et al. found that [38]*,* most miombo had been heavily disturbed because of local benefits attached to them like dry-season fodder for large livestock populations, fuelwood for domestic use and rural industry and construction materials for farm structures and homes for millions. Higher AGC removals in miombo woodlands due to fire is because of its roles as the management tools. When fire is frequently and uncontrolled, it could kill trees and eventually cause carbon emissions.

Considering administrative zones, charcoal and timber account for higher AGC removals in the Eastern zone. Conversely, charcoal and firewood collection account for higher number of stem removals in this zone. This is due to the highest charcoal and timber consumption that may be linked to the closeness to Dar es Salaam city. Dar es Salaam, Tanzania’s largest city, accounts for more than 50% of all charcoal consumed in the country [39]. Moreover, higher timber consumption in this zone could be attributed to high demand of timber for furniture and infrastructure development particularly houses in the Dar es Salaam city. Dar es Salaam is the primary destination of timber and timber products (including all round and sawn timber) and accounting for 87% of timber felled in southeast Tanzania [40]. Other important domestic markets of timber and wood products from the zone are Zanzibar, Mafia and Arusha [29]. Shifting cultivation and charcoal account for the largest number of stems and AGC removals in the northern zone probably due to intensification of shifting cultivation. In the lake zone, fire, firewood collection and pole account for the large stems and AGC removals probably due to heavy dependence trees for cooking energy and constructions purposes. Furthermore, presence of dry litter that foster fire occurrence explains the removals due to fire in this zone. The regular fires in the miombo region can, if too frequent or intense, cause mortality of large and small trees and prevent regeneration [41]. Likewise, long-term plot-scale experiments had shown that under annual burning miombo woodlands are converted to grassland [41, 42], and that in the absence of fire, miombo starts to form closed canopy forest [41].

Regarding vegetation types, shifting cultivation, charcoal, timber poles, and firewood collection accounted for the highest AGC and number of stems removals in the woodland with scattered woodland. Shifting cultivation type of farming in the country is practiced by more than 70% of the population. Other scholar [29] found that shifting type of agriculture is common and practiced for all annual crops grown in Tanzania. The most cited reasons for shifting their plots are; invasion of weeds and evading wild animals. On the other hand, natural death, timber and shifting cultivation account for the largest AGC removals in the closed woodland. Natural death is more prominent in this vegetation probably because protection forest and wildlife area comprise most of this vegetation where no harvesting is allowed. Regarding timber, most of the timber is removed illegally.

In terms of ownership types, fire, timber charcoal and natural death account for higher number of stems removals in all the categories of ownership. This may be attributed to population growth and inadequate presence of alternative sources of energy for cooking and construction purpose that ultimately forces people to heavily depend on charcoal and timber. Irrespective of the fact that, forest under general land is almost open access in which free movement of people take products [43], its contribution to the total removals is low as opposed to private and village land. On the other hand, shifting cultivation accounts for the highest AGC removals in the shifting agriculture and agricultural land probably because shifting cultivation type of agriculture characterize the ownership types.

Considering land use types that miombo woodlands falls, it was revealed that shifting cultivation and charcoal account for the highest number of stem removals in grazing and shifting cultivation land. This is because large numbers of stems are removed during land preparation in the shifting cultivation. Likewise, charcoal making and firewood collection characterize the land. Furthermore, natural death, poles, charcoal and firewood collection caused more stems cut in the production forest, protection forest and wildlife reserves land. This is much explained by the nature of the ownership types and the large dependence of charcoal and firewood for cooking energy while poles for construction purposes. In contrast, AGC removals that ultimately end up into carbon emissions are driven by charcoal, natural death, shifting cultivation, poles, timber, fire and firewood collection in all land use types. This may be attributed by population growth that demand more products from the woodlands and climate change impacts that naturally kill trees through eruption of diseases and drought. Moreover, economic growth based on the export of primary commodities and an increasing demand for timber and agricultural products in a globalizing economy are critical reasons behind carbon emissions.

## Conclusion

AGC removals in miombo woodlands of Mainland Tanzania are caused by a range of drivers that lead to varying levels of carbon emissions. The results revealed that charcoal, timber, shifting cultivation, fire, firewood collections, poles and natural death are the prominent main drivers of AGC removals in mainland Tanzania. Furthermore, charcoal, shifting cultivation and fuelwood caused higher number of tree removals as opposed to timber, natural death and fire that accounted for the highest AGC removals. For the purpose of reducing stems and AGC removals in the entire miombo woodlands and its subsequently categories; all drivers of removals i.e. fires, firewood collection, grazing by both wildlife, domesticated animals, carving, poles, shifting cultivation, timber, and mining should be managed. However, the management priorities should consider the significance contribution by charcoal, shifting cultivation and fuelwood for stem removals and timber, natural death and fire for AGC removals. This would contribute to creation of considerable carbon sink as well as ensure persistent potential for the miombo woodlands to store carbon thus contributing to the REDD+ process in Tanzania. Moreover, this kind of study need to be conducted in other vegetation types like Montane and Mangrove forest in Tanzania. On the other hand, since NAFORMA data provide national picture on drivers and their variation on AGC removals, we recommend site specific studies be conducted to bring information that would be used to devise appropriate strategies to deal with drivers in their order of contribution to AGC removals in the local settings. Additionally tree planting for timber and energy should be encouraged as mitigating measure.

## Methods

### Study area description

**Fig. 1:**
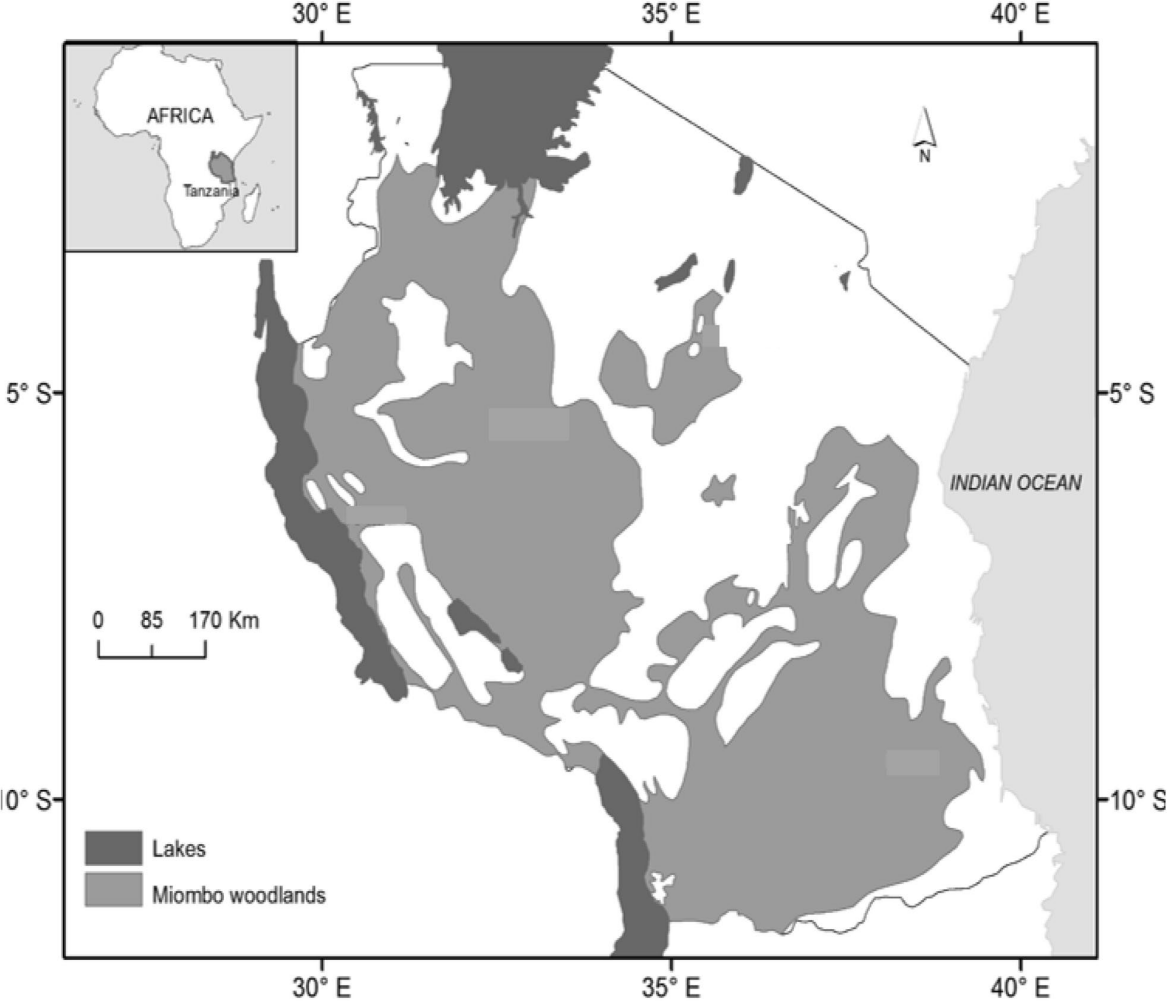
A map of mainland Tanzania showing Miombo woodlands (modified from [45])

The study involved the entire miombo woodlands of mainland Tanzania that covers about 44.7 million ha (Fig. 1). Vast areas of miombo woodlands falls under the village lands ownership, which lack proper management institution [44]. Depending on altitude and latitude, mainland Tanzania is characterised by both tropical and subtropical climates. The mean annual rainfall varies from below 500 to over 2000 mm per annum. The rainfall for the large part of the country is bimodal with short rains from October to December and long rains from March to May. The weather conditions of the country may be divided into a hot dry season from mid-August to the end of October, a hot wet season from November to the beginning of April and a relatively cool dry season from April to mid-August.

## Data collection

**Fig. 2:**
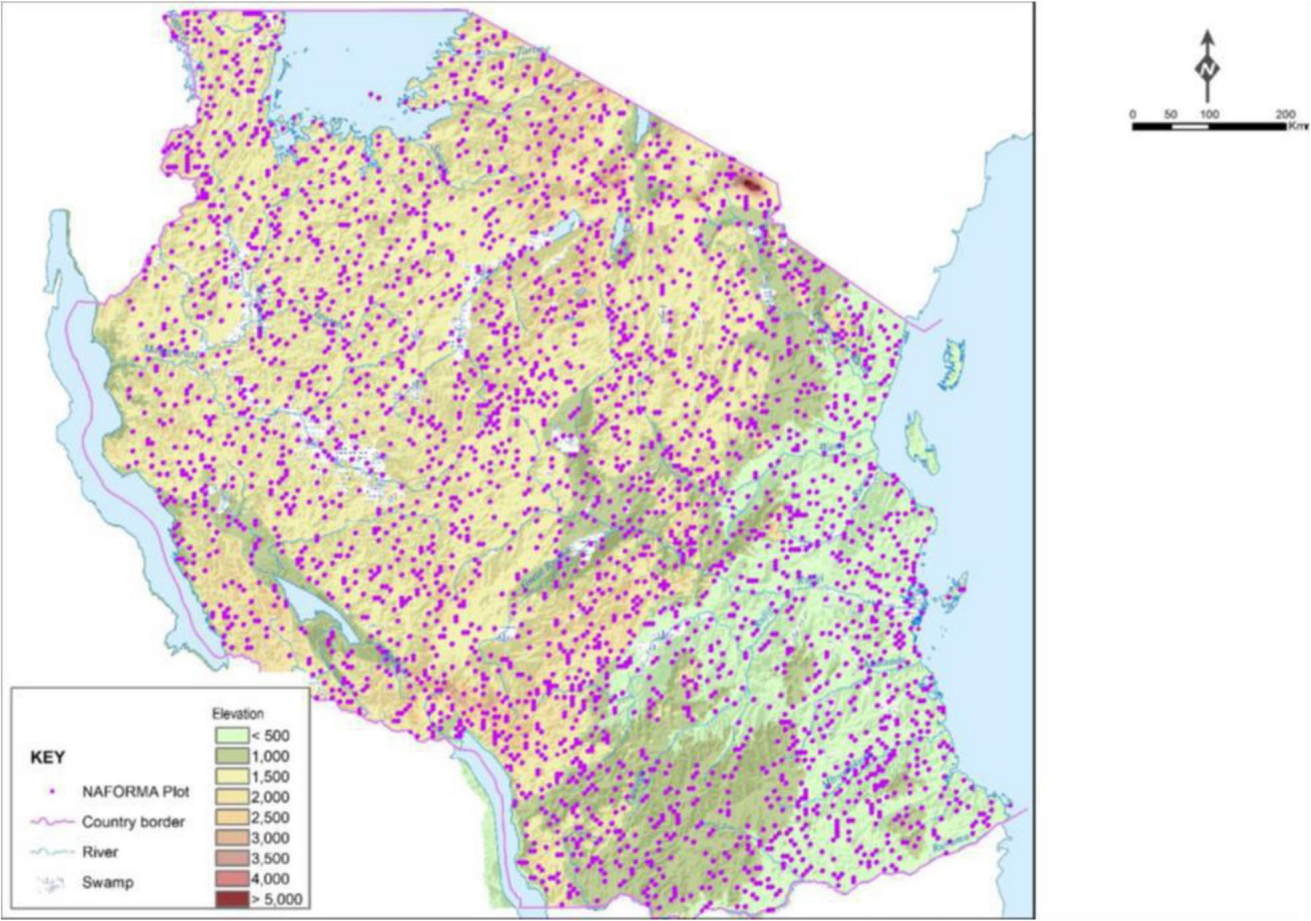
Distribution of sample plots in Mainland Tanzania (source: [2])

The data used for the assessment of drivers and their influence on variation of AGC removals presented in this paper were collected by NAFORMA [26]. Systematic double sampling for stratification with optimal allocation of individual plots in cluster was sampling design of the NAFORMA. The design was chosen after sampling simulations to reduce uncertainty of estimates under given budget constraints. The data were collected in a total of 32 660 field plots established across all land cover types in mainland Tanzania (Fig. 2). The detail of the sampling design, data collection and other uncertainties are given in [2, 3, 26, 46, 47].

### Data acquisition

For the purpose of this study, all plots that were surveyed for stumps measurement were extracted from NAFORMA database. During extraction, the whole NAFORMA data set was imported to R software. By using “sqldf” R package which runs Structured Query Language (SQL) statements on R data frames, all stump data from miombo woodlands were extracted. Finally, data cleaning i.e. removal of noisy data was done. A total 7323 stumps from 16803 plots were extracted.

## Data analysis

### Analysis of drivers of aboveground carbon removals

To obtain the drivers of AGC removals, the identified trees with their corresponding drivers for their removals were listed. The drivers were sorted alphabetically in order to identify total number of drivers responsible for removals in miombo woodlands. Those drivers that were similar like removals due to firewood collection for domestic and industrial use were regarded as firewood collection.

### Drivers and their influence on aboveground carbon removals

We included multiple drivers identified (11 drivers) in the analysis, so that the interrelationships between the drivers and AGB removed could be accounted. Since each tree had driver of its removal, driver and their influence on AGC removals was estimated. Removed AGB per tree was estimated using allometric equation i.e. AGB = *a* × (*SD*)^*b*^. This equation calculate biomass of a tree from stump diameter only [7]. The estimated individual tree AGB removal and its corresponding driver was divided by age of the stump to get the rate of AGB removals per year. Then, AGB removals value per year per tree was multiplied by 0.49 as the conversion factor of AGB to AGC [25]. AGC removals per year per tree was summed up and expressed on per plot basis. Since each stratum had unique sampling intensity, calculation of expansion factors (*EF*) for each respective stratum was inevitable since simple mean AGB would ignore the nature of the sampling design upon which the data were collected. The *EF* describes the area in which a sample plot represents in each stratum. Since first phase sampling units were distributed proportionally to stratum area, the area of the stratum *p* (${\widehat{A}}_{p}$) was calculated as follows:

1 $${\widehat{A}}_{p}=A*\frac{{n}_{p}}{{n}_{1}}$$

where n_p_ is number of first phase plots in stratum *p* (ha); n_1_ is total number of first phase plots; and A is total inventory area (Mainland Tanzania area). Practical sequences of computation are shown below and further described in Tomppo et al*.* [2, 3].

2 $$E{F}_{p}=\frac{{\widehat{A}}_{p}}{{n}_{p}}$$

where EF_p_, is plot expansion factor of stratum p, Âp is area of stratum p; and *n*_*p*_ total number of plots observed in stratum p.

Consider *n*_*t,p*_ number of plots of landcover sub-class *t* falling in stratum *p.* The area *Â*_*t,p*_ of landcover sub-class *t* in stratum *p* was computed as:

3 $${\widehat{A}}_{tp}=\sum_{t\varepsilon p}{n}_{t{p}^{*}}E{F}_{p}$$

where *n*_*t,p*_ number of plots of land cover sub-class *t* in stratum *p;* and *EF*_*p*_ is Expansion Factor of stratum *p.*

Area of land cover sub-class *t* in the country is the summation of areas of land cover sub-classes *t* found in each stratum, i.e. *Â*_*t*_ = *Â*_*t1*_ + *Â*_*t2*_ + *Â*_*t3*_ + ...... *Â*_*tp*_ where *tp* is land cover sub-class *t* in stratum p. Consequently, AGC plot level values were multiplied by respective *EF* value corresponding to each stratum. The AGC plot level values were expressed on per hectare (ha). Moreover, to obtain the influence of each driver on AGC removals per ha, AGC per year per ha were summarized into their corresponding drivers. Lastly, AGC per year per ha and their drivers were summarized based on zones, miombo vegetation subtypes, Land use and Ownership types.

To explore if there were differences in mean volume and carbon estimates across categories of miombo woodlands, statistical significance test was used. Two-way analysis of variance (ANOVA) were used. Analysis of mean volume and carbon across categories was then done using Duncan’s multiple range test for ratio to explore which means are different.

## Data Availability

All authors declare that the datasets used in this study are available upon request from the Tanzania Forest Service, Ministry of Natural Resources and Tourism Tanzania.

## Abbreviations

AGC: Aboveground carbon
NAFORMA: National Forest Resources Assessment and Monitoring
GHG: Greenhouse gas emissions
AGB: Aboveground biomass
NFI: National forest inventory
REDD+: Reduced Emissions from deforestation and forest degradation “plus,” the role of conservation, sustainable management of forests, and enhanced carbon stock
TFS: Tanzania Forest Services Agency
IPCC: Intergovernmental Panel for Climate Change
